# A High-Accuracy Calibration Method for a Telecentric Structured Light System

**DOI:** 10.3390/s22176370

**Published:** 2022-08-24

**Authors:** Chao Chen, Ya Kong, Huaiwen Wang, Zonghua Zhang

**Affiliations:** 1 Tianjin Key Laboratory of Refrigeration Technology, Tianjin University of Commerce, Tianjin 300134, China; 2 School of Mechanical Engineering, Tianjin University of Commerce, Tianjin 300134, China; 3 Tianjin Xunlian Technology Co., Ltd., Tianjin 300308, China; 4 School of Mechanical Engineering, Hebei University of Technology, Tianjin 300130, China

**Keywords:** telecentric structured light system, system calibration, fringe projection, 3D measurement

## Abstract

We propose a method for accurately calibrating a telecentric structured light system consisting of a camera attached to a bilateral telecentric lens and a pin-hole projector. The proposed method can be split into two parts: axial calibration and transverse calibration. The first part is used for building the relationship between phase and depth by means of a planar plate with ring markers on its surface at several different positions in the measuring volume. The second part is used for establishing the relationship between transverse coordinates and pixel positions with the depth offered by a translation stage and the extracted ring centers. Compared with existing methods that require projector calibration, the proposed method can avoid a propagation of the correspondence error between the camera imaging plane and projector imaging plane, thus increasing calibration accuracy. The calibrated telecentric structured light system is further used for three-dimensional (3D) reconstructions of a planar, a ruled surface, and complex surfaces. Experimental results demonstrate that the proposed system calibration method can be used for accurate 3D measurement.

## 1. Introduction

With the development of the micro manufacturing technology, there has been an increasing demand for the research of high-accuracy micro-level three-dimensional (3D) measurement methods. A classical structured light technology using a camera with an ordinary lens and a projector is taken as a probable solution for micro-scale 3D profilometry because of its advantages of non-contact operation, full-field acquisition, and high accuracy. To apply this technology to micro-scale measurement, the researchers replaced an ordinary lens with a telecentric lens to form telecentric structured light systems, which make use of the advantages of the telecentric lens with constant magnification over a specific volume, nearly zero distortion, and increased depth of field (DOF) [1,2]. According to the relative position of the optical axes of projector and camera, these systems can be grouped roughly into two types, coaxial or parallel-axes systems and crossed-axes systems.

In the first kind of system, the optical axes of a projector and a camera are set to coaxial or parallel for increasing the measuring range of the DOF based on the orthogonal projection characteristics of a telecentric camera [3,4,5,6]. A series of circle phase-shifted fringe patterns are projected and captured for calculating phase maps and extracting zero-phase point coordinates. With the aid of point coordinates, the 3D shape of an object surface can be reconstructed with the geometry of projected and captured light rays. However, such systems are susceptible to the zero-phase point detection problem because the zero-phase point may be out of the field of view (FOV) of the camera, which inevitably introduces additional geometrical errors between camera and projector. Recently, Zhong et al. [7] designed a dual-telecentric structured light system using the modulation distribution, instead of phase information, for 3D reconstruction. Therefore, it is suitable for measuring an object surface with shutoff or steep slope to solve the problems of shadow and occlusion.

In the other kind of system, such as classical structured light systems, the triangular relationship formed by a projector, a telecentric camera, and a measured object is used for 3D measurement. The calibration of such systems, which aims to determine the intrinsic and extrinsic parameters of a camera and a projector, is a challenge because telecentricity leads to insensitivity of depth altering in the optical axis of camera. To deal with this challenge, several system calibration methods have been proposed for accurately measuring the 3D topography of an object surface. For instance, Li et al. [8] proposed a method to calibrate a telecentric camera and a projector simultaneously by using horizontal and vertical sinusoidal fringe sequences. Then, the 3D shape of a measured object can be reconstructed with the obtained intrinsic and extrinsic parameters of the camera and the projector. In their method, the one-to-one mapping between camera pixels and projector pixels needs to be established for calculating the projector parameters based on phase maps resulting from captured fringe images in the vertical and horizontal directions. Later, some researchers either changed the calibration methods [9,10,11,12] of the telecentric camera or used the binary defocusing technique [13] to improve the measurement accuracy. The main drawback of such methods is that the correspondence accuracy between a camera imaging plane and a projector imaging plane directly influences the performance of projector calibration; there will no doubt be a propagation of the correspondence error. Recently, Pistellato et al. [14] used a sphere with a known radius as calibration target for calibrating a telecentric camera–projector setup. However, the method is not suitable for a telecentric structured light system consisting of a telecentric camera and a pin-hole projector.

To overcome the above problems, we propose a polynomial-based method to calibrate a telecentric structured light system without requiring projector calibration. The proposed method involves two transformations. One is from pixel coordinates to transverse coordinates. The other is from phase information to depth data. The former can be accomplished by invoking the orthogonal projection model of a telecentric camera. The latter can be achieved by using vertical fringe sequences and a translation stage. Compared with existing calibration methods that need to calibrate a projector with the help of a camera, the proposed method can not only increase measurement accuracy, but also avoid the complex process of projector calibration. In the following sections, the principle of the proposed system calibration method is first described. Then, the effectiveness and accuracy of this method are validated by 3D shape measurements of a white plate with ring markers at different positions and orientations, a spherical object with known diameter size, and two objects with complex surfaces. Finally, the contributions of our work and remaining problems to be solved in future research are summarized.

## 2. Principle

With the assistance of a planar plate with equally spaced ring markers on it, the calibration of a telecentric structured light system consisting of a telecentric camera and a pin-hole projector needs to perform two transformations. One is from phase to depth, which is called axial calibration. The other is from pixel positions to transverse coordinates, which is called transverse calibration. Prior to system calibration, the intrinsic and extrinsic parameters of a telecentric camera can be determined from ring centers extracted by the ellipse fitting algorithm [15,16].

### 2.1. Axial Calibration

**Figure 1:**
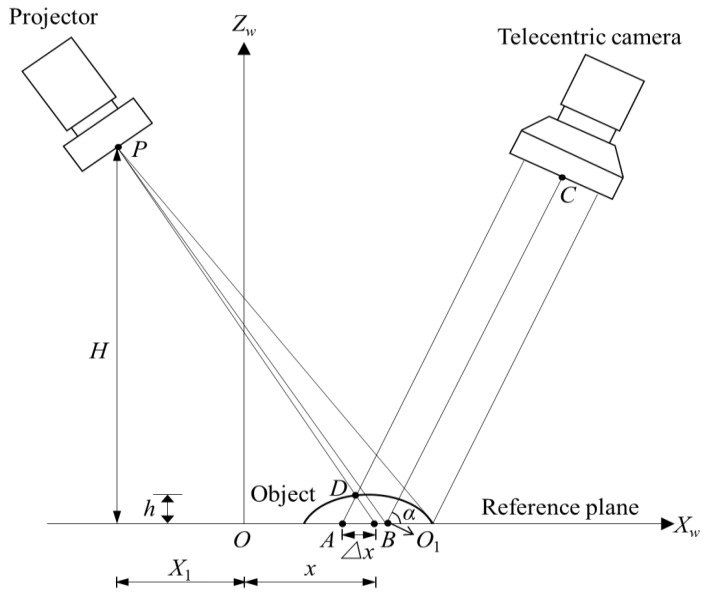
Imaging model of a telecentric fringe projection system.

Figure 1 shows the imaging model of a telecentric structured light system, where the optical axis of the projector *PO*_1_ crosses the optical axis *CO*_1_ of the camera at point *O*_1_ on the reference plane and the *Z_w_* axis is perpendicular to the reference plane. *H* is the distance from the projection center *P* to the reference plane. *D* is an arbitrary point on the measured object. The points *A* and *B* denote the effects of fringe deformation owing to the presence of the object rather than the reference plane. In other words, a sinusoidal fringe pattern originally projected at point *B* of the reference plane is now projected at point *D* on the object surface and then reflected along ray *AD* to the camera imaging plane. According to the geometry, the relationship between Δ*x* and deformation *h* can be expressed by

(1) $$h=\frac{H\tan\alpha }{{H+(x+X}_{1})\tan\alpha }∆x\;$$

where *α* is the angle between the optical axis of the telecentric camera and the reference plane. The phase difference ∆φ between the object surface and the reference plane is

(2) $$∆{\varphi =2\pi f}_{0}∆x$$

Equation (1) can be rewritten as follows:

(3) $$h=\frac{H∆\varphi \tan\alpha }{\left[{H+(x+X}_{1})\tan\alpha \right]{2\pi f}_{0}}$$

where *f*_0_ is the spatial frequency of the fringe pattern projected on the plane normal to the projector optical axis. In theory, these system parameters, *H*, *α*, *x*, *X*_1_, and *f*_0_, can be obtained. However, the direct calibration of these parameters is extremely difficult and complicated in practice. The polynomial calibration model used for the calibration of traditional structured light systems [17,18] is more flexible than other models for allowing the system components to be arbitrarily arranged. Therefore, we introduce the polynomial model to the calibration of a telecentric structured light system for establishing the relationship between phase and depth.

**Figure 2:**
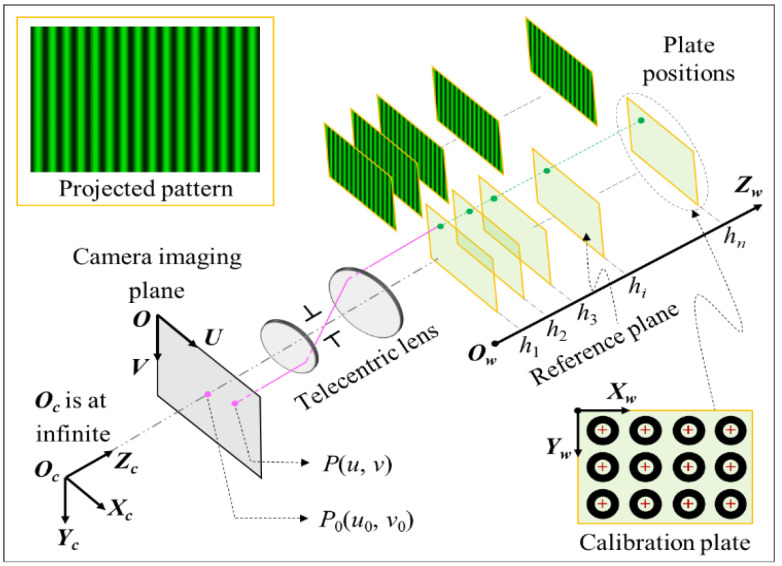
Schematic diagram of the proposed system calibration method.

As a matter of fact, Equation (3) for every point (*u*, *v*) of the calibration volume can be expressed by the following polynomial equation [16]:

(4) $$∆h(u,\;v)=\overset{N}{\underset{n=0}{\sum }}a_{n}(u,\;v)\;∆{\varphi (u,\;v)}^{n}$$

where *a_n_*, *n* = 0, …, *N* are the polynomial coefficients, which contain the system parameters. Consequently, a Look-Up Table (LUT) for *a_n_* is required to be constructed at each pixel position for establishing the relationship between phase and depth. Axial calibration aims to calculate the coefficients of the polynomial equation. The plate with ring markers on it is located at several parallel positions *h_i_* in measuring volume with a translation stage, as shown in Figure 2. At each plate position, sinusoidal fringe patterns are projected on the plate surface for offering the phase information of each pixel. It is noted that the depth ∆h (u, v) in Equation (4) is a relative depth value with regard to the reference plane, so the depth data offered by the translation stage needs to be transformed into the reference coordinate system, whose origin is approximately in the middle of the measuring volume.

### 2.2. Transverse Calibration

Transverse calibration needs to establish the relationship between pixel coordinates and X, Y coordinates. This relationship is linear in an actual telecentric structured light system because distortion of the telecentric lens can be ignored and the required depth information is offered by a precise translation stage. Then, the transverse calibration can be described by the following polynomial equations:

(5) $$\left\{\begin{array}{c} x_{w}{=b}_{1}{(u,\;v)h+b}_{0}\left(u,\;v\right)\\ y_{w}{=c}_{1}{(u,\;v)h+c}_{0}\left(u,\;v\right) \end{array}\right.$$

where *b*_0_, *b*_1_, *c*_0_, and *c*_1_ are the polynomial coefficients, which depend on the chosen position (*u*, *v*). (*x_w_*, *y_w_*) are the coordinates of a point on the plate in the reference coordinate system, which can be obtained by the following two steps. Therefore, an LUT for *b_i_* and *c_i_*, *i* = 0, 1, can be constructed at each pixel position for establishing the relationship between the transverse coordinates and pixel positions.

Step #1: Calculating the spatial position relationship between the plate and camera imaging plane. As shown in Figure 2, the projection from an arbitrary ring center on each plate to the camera imaging plane can be determined by [19]

(6) $$\left[\begin{array}{c} u_{c}\\ v_{c}\\ 1 \end{array}\right]=\left[\begin{array}{ccc} \alpha & 0 & u_{0}\\ 0 & \beta & v_{0}\\ 0 & 0 & 1 \end{array}\right]\left[\begin{array}{ccc} r_{11} & r_{12} & t_{1}\\ r_{21} & r_{22} & t_{2}\\ 0 & 0 & 1 \end{array}\right]\left[\begin{array}{c} x_{\mathrm{wc}}\\ y_{\mathrm{wc}}\\ 1 \end{array}\right]=H\left[\begin{array}{c} x_{\mathrm{wc}}\\ y_{\mathrm{wc}}\\ 1 \end{array}\right]$$

where *α* and *β* are respectively the magnification radio along the *U* and *V* axes of the imaging plane, (*u*_0_, *v*_0_) are the center coordinates of the camera imaging plane, *r_ij_* and *t_i_* respectively represent elements of the 3 × 3 rotation matrix and the 3 × 1 translation vector, (*x_wc_*, *y_wc_*) are the world coordinates of teh ring centers, (*u*_c_, *v*_c_) are the corresponding pixel coordinates on the imaging plane, and **H** is a 3 × 3 homography matrix between the plate and the camera imaging plane.

Step #2: Calculating the world coordinates of each pixel point on the plate. The world coordinates of each pixel point on the plate can be calculated by performing a reverse operation on Equation (6):

(7) $$\left[\begin{array}{c} x_{w}\\ y_{w}\\ 1 \end{array}\right]{=H}^{-1}\left[\begin{array}{c} u\\ v\\ 1 \end{array}\right]$$

where (*x_w_*, *y_w_*) are the world coordinates of each pixel point on the plate, (*u*, *v*) are the corresponding pixel coordinates on the camera imaging plane, and **H**^−1^ is the inverse matrix of **H**.

## 3. Experiments and Results

### 3.1. Experimental System

**Figure 3:**
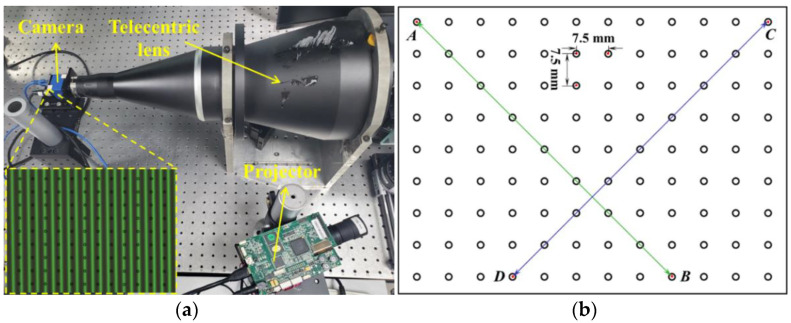
(**a**) Photograph of the experimental system and (**b**) illustration of the measured distances on the calibration plate.

The photograph of the established telecentric structured light system includes a projector (PRO4500, Texas Instruments, Texas, United States) with a resolution of 912 × 1140 pixels and a camera (ECO445CVGE, SVS-VISTEK GmbH, Innsbruck, Germany) with a resolution of 1296 × 964 pixels and a pixel size of 3.45 µm, as shown in Figure 3a. The bilateral telecentric lens mounted with the camera has the model number of GCO230105 with a magnification of 0.057. Additionally, a calibration plate with 12 × 9 discrete ring markers was used for system calibration and accuracy evaluation, as shown in Figure 3b. The distances of each adjacent markers in the horizontal and the vertical directions have the same value of 7.5 mm. For all experiments, a four-step phase-shifting algorithm was used for calculating the wrapped phase and the optimum fringe numbers selection method [20] was used for calculating the unwrapped phase at each pixel position.

### 3.2. System Calibration Procedure

According to the principle of the proposed system calibration method in Section 2, the whole calibration procedure is performed step by step as follows.

**Figure 4:**
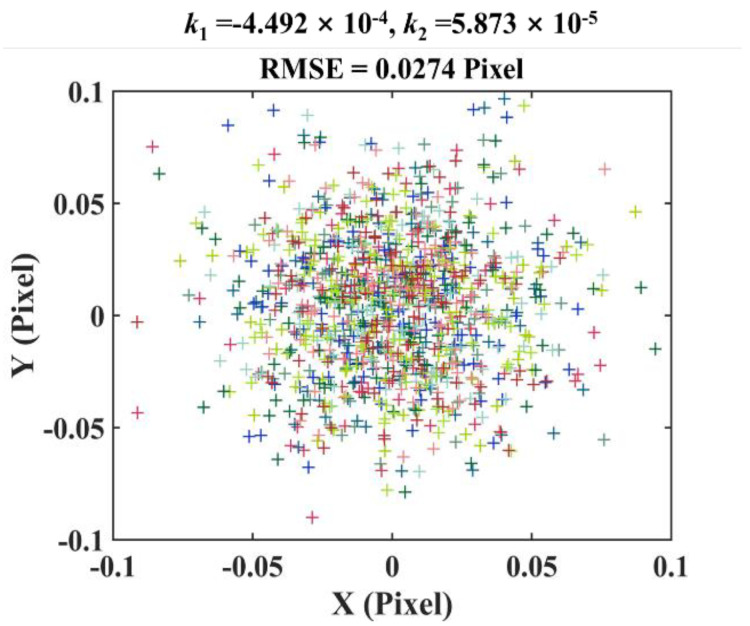
Re−projection errors of the telecentric camera.

Step #1: Telecentric camera calibration before system calibration. The calibration plate was firstly placed at ten different positions and directions in the measuring volume. At each position, the plate image was captured and then used for extracting the ring centers. Finally, the intrinsic and extrinsic parameters of the telecentric camera were determined through the correspondences between the pixel coordinates of the extracted marker points and their spatial coordinates. The camera calibration accuracy can be assessed on the basis of the root-mean-square error (RMSE) of the ring centers, which can be calculated by the following equation [21]:

(8) $$\mathrm{RMSE}=\frac{\sum_{i=1}^{N}\sqrt{\left[{\left(u_{c}-\hat{u_{c}}\right)}^{2}+{\left(v_{c}-\hat{v_{c}}\right)}^{2}\right]}}{N}$$

where *N* is the total number of the ring centers, $\left(u_{c},\;v_{c}\right)$ are the extracted ring center locations, and $\left(\hat{u_{c}},\;\hat{v_{c}}\right)$ are the re-projection point locations, which were calculated from the calibrated camera parameters. The re−projection errors of the calibrated camera are calculated for the performance of camera calibration, as shown in Figure 4. In the experiment, the RMSE = 0.0274 pixels is small, which proves that the telecentric camera is calibrated successfully. Additionally, we notice that the calculated radial distortion coefficients *k*_1_ = −4.492 × 10^−4^ and *k*_2_ = 5.873 × 10^−5^ are very small, which, in fact, is consistent with the low-distortion property of telecentric lens. Normally, other types of distortions such as tangential and prism distortions are much smaller than the radial distortion. Therefore, it is reliable that we ignore lens distortion in system calibration.

Step #2: Capture of fringe images and texture images for system calibration. During the system calibration, the same plate was strongly fixed on the translation stage (HGAM307, Henggong Instrument Co., Ltd., Beijing, China) with a resolution of 10 μm and then translated along the depth direction from −6 to 6 mm, with an increment of 1 mm between successive positions. At each position, three sinusoidal fringe pattern sets with optimum fringe numbers of 100, 99, and 90 and each set with four phase-shifted fringe patterns with π/2 shift in between were projected on the plate surface. The camera captured the twelve fringe images for calculating the phase information and one texture image of the plate under white illumination for extracting the ring centers. These images and depth locations were saved for subsequent processing.

Step #3: Axial calculation. Using the captured fringe images, the unwrapped phase of all the pixels for each plate position was first calculated. Then, the plate position in the middle of the measuring volume, i.e., *Z_w_* = 0, was selected as a reference plane to transform both the saved depth locations and the acquired phase into the reference coordinate system. Note that when the relative phase and the relative depth information are known, an LUT for *a_n_* at each pixel can be calculated and saved by invoking Equation (4) for the following reconstruction of depth data. It should be mentioned that a fifth fitting was chosen for axial calibration to achieve the optimal phase resolution.

Step #4: Transverse calibration. Using the captured texture image of the plate, the center position of each marker was first extracted. Afterward, based on Equation (7), the transverse coordinates of all pixel points at each plate were calculated with the obtained intrinsic and extrinsic parameters of the camera in Step #1. The pixel coordinates and the transverse coordinates on the plate from the ten images were used to construct an LUT for *b*_0_, *b*_1_, *c*_0_, and *c*_1_ at each pixel according to Equation (5). All the obtained coefficients were saved for the following reconstruction of transverse data.

### 3.3. Quantitative and Qualitative Evaluation

To evaluate the performance of the proposed system calibration method, we also calibrated the system by using the method requiring projector calibration [8]. The plate was exactly placed in spatial positions used in the above camera calibration method to ensure consistent camera parameters. At each position, twelve vertical and twelve horizontal sinusoidal fringe patterns with the same optimum fringe numbers were projected onto the plate surface. The telecentric camera captured the deflected fringe images for calculating phase maps in the vertical and horizontal directions and a texture image under white illumination for extracting the center positions of all markers. We used the obtained phase maps in two directions for calculating the corresponding points of all markers in the projector pixel coordinate system. By establishing the correspondence between the pixel coordinates of the marker points and their spatial coordinates, the intrinsic and extrinsic parameters of the projector were determined. Eventually, the intrinsic and extrinsic parameters of the system were obtained and could be used for 3D measurement. It should be noted that the two methods used the same reference position to ensure that the reconstructed 3D data has the same coordinate system.

For accuracy comparison, we captured an additional set of five different poses in the calibration volume and measured the distances of the green line $\bar{\mathrm{AB}}\;$ and the distances of the blue line $\bar{\mathrm{CD}}\;$ shown in Figure 3b. The two lines are formed by the ring center A, B, C, and D and the distances of two lines are 84.853 mm theoretically. We used the proposed calibration method and the method using projector calibration for reconstructing the 3D shape of the plate at each position, and then extracted the 3D coordinates of these four points. The Euclidean distances $\bar{\mathrm{AB}}$ and $\bar{\mathrm{CD}}$ were calculated and are listed in Table 1. By comparing the difference between the measured and actual values, we calculated that the mean errors with the proposed method are less than those with the method using projector calibration. The results evidently prove the effectiveness of the proposed system calibration method.

**Figure 5:**
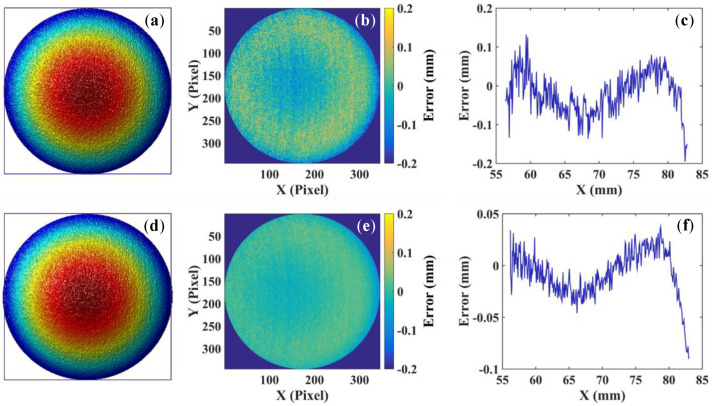
Measurement results of a spherical surface with two methods. (**a**–**c**) Results with the method requiring projector calibration: (**a**) 3D geometry; (**b**) 2D error map of (**a**); (**c**) cross section of the middle line from (**b**). (**d**–**f**) Results with our method: (**d**) 3D geometry; (**e**) 2D error map of (**d**); (**f**) cross section of the middle line from (**e**).

To further evaluate the calibration accuracy, we reconstructed a regular sphere with a diameter of 38.1 mm using both the method requiring projector calibration and our method. The reconstructed 3D geometries are shown in Figure 5a,d, respectively. Then, we fitted the reconstructed 3D result to an ideal sphere. The model of the ideal sphere is (*x* − 68.59)^2^ + (*y* + 1.99)^2^ + (*z* + 560.32)^2^ = 362.14 with the method requiring projector calibration, and (*x* − 68.50)^2^ + (*y* + 1.90)^2^ + (*z* + 560.14)^2^ = 362.71 with our method. we compared the reconstructed 3D geometry with the ideal sphere. The corresponding 2D error maps are shown in Figure 5b,e. Finally, we extracted the error distribution of the middle line from Figure 5b,e and plotted them in Figure 5c,f. The mean error and standard deviation are 0.0429 mm and 0.0048 mm with the method requiring projector calibration and 0.0182 mm and 0.0034 mm with our method. These results again demonstrate that the proposed system calibration method has better performance in calibration accuracy than the method requiring projector calibration.

**Figure 6:**
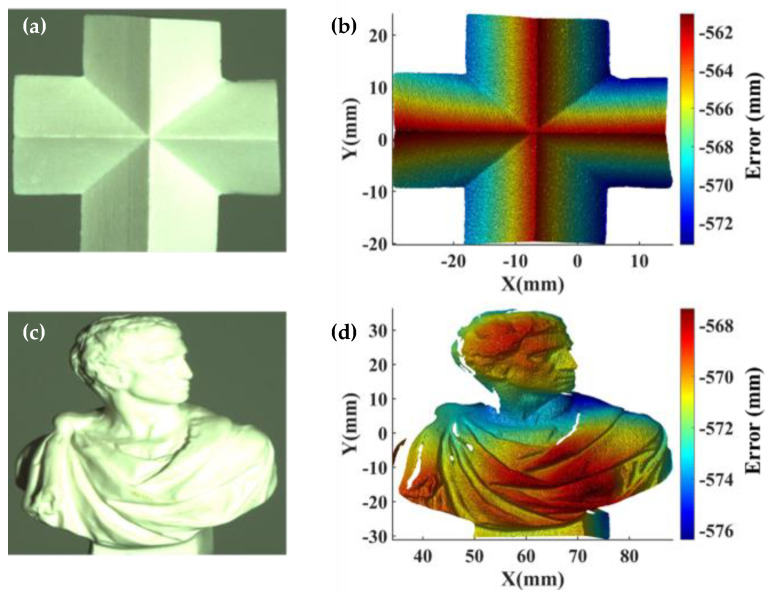
3D geometries with the proposed system calibration method. (**a**,**c**) Photographs; (**b**,**d**) reconstructed geometries.

To visually evaluate the performance of the proposed system calibration method, we measured two plasters having freeform surfaces as shown in Figure 6a,c. Specifically, twelve sinusoidal fringe patterns with the four-step phase shifting and the fringe numbers of 100, 99, and 90 through the green channel were projected on the plasters’ surfaces by the projector. Then, the fringe patterns reflected on the plasters’ surfaces were captured by the telecentric camera from another viewpoint. Subsequently, the unwrapped phase at each pixel was calculated by the four-step phase-shifting algorithm and the optimum three-fringe number selection method. By transforming the obtained absolute phase maps into depth and transverse data, we reconstructed the 3D geometries of the plasters, as shown in shown in Figure 6b,d. It is clear that the details of the plasters were perfectly reconstructed. This experiment demonstrated that the proposed calibration method can supply high-quality 3D shape reconstruction for objects with freeform surfaces. 

## 4. Conclusions

We have proposed a polynomial-based calibration method for a telecentric structured light system, which consists of a telecentric camera and a pin-hole projector. The proposed method is divided into axial calibration and transverse calibration. The former needs to build up the relationship between the absolute phase and the depth data. The latter requires establishing the relationship between pixel positions and X, Y coordinates. Compared with the existing methods that need to calibrate the projector, the calibration method proposed in this research for telecentric structured light systems has the following advantages:

(1) Accuracy. The proposed method averts the coupling of correspondence errors between camera pixels and projector pixels, hence increasing measurement accuracy.
(2) Ease of operation. During the whole calibration process, a calibration plate is fixed on a translation stage and then successively translated along the depth direction. The orientation of the plate does not change, so the proposed calibration method is easy to operate.
(3) Simplicity. The proposed calibration method avoids projector calibration, which makes calibration simple.

In the proposed method, applying more calibration plate positions can cover the measuring volume better. This can provide a higher calibration accuracy, but increases time consumption to perform projection and capture of fringe patterns. To keep a balance between calibration accuracy and time complexity, the appropriate number of calibration plate position should be chosen for system calibration according to the depth of a measured object. Additionally, a translation stage is required to accurately provide the calibration plate with known depth information to meet the requirement of high accuracy measurement, so the calibration procedure is difficult to perform out of the laboratory environment. Comprehensively considering the pros and cons, we deduce that the proposed method is quite suitable for high-accuracy 3D measurements of small-scale objects [22] and has great potential in 3D shape measurement of micro-parts with a size on the order of millimeters, used in micro-parts mechanical system (MEMS) [23].

## Figures and Tables

**Table 1:** Measurement results of two lines on the calibration plate.

Proposed Method				
**Pose No.**	$\bar{\mathrm{AB}}$ **(mm)**	**Error (mm)**	$\bar{\mathrm{CD}}$ **(mm)**	**Error (mm)**
1	84.896	0.043	84.899	0.046
2	84.879	0.026	84.881	0.028
3	84.838	−0.015	84.887	0.034
4	84.885	0.032	84.899	0.046
5	84.830	−0.023	84.832	−0.021
Mean	84.866	0.013	84.880	0.027
**Method Using Projector Calibration**				
**Pose No.**	$\bar{\mathrm{AB}}$ **(mm)**	**Error (mm)**	$\bar{\mathrm{CD}}$ **(mm)**	**Error (mm)**
1	84.911	0.058	84.903	0.050
2	84.834	−0.019	84.901	0.048
3	84.891	0.038	84.869	0.016
4	84.843	−0.010	84.885	0.032
5	84.899	0.046	84.879	0.026
Mean	84.875	0.023	84.887	0.034

## Data Availability

Not applicable.
